# Equity implications for sanitation from recent health and nutrition evidence

**DOI:** 10.1186/s12939-017-0709-5

**Published:** 2017-12-06

**Authors:** A. A. Cronin, M. E. Gnilo, M. Odagiri, S. Wijesekera

**Affiliations:** 1 Chief Water, Sanitation and Hygiene (WASH) program, UNICEF, World Trade Centre Block 6 (10th Floor), Jalan Jenderal Sudirman Kav. 31, Jakarta, 12920 Indonesia; 2 Sanitation and Hygiene Specialist, Water, Sanitation and Hygiene Section, UNICEF, 3 UN Plaza, New York, NY 10017 USA; 3 Water and Sanitation Officer, UNICEF, World Trade Centre Block 6 (10th Floor), Jalan Jenderal Sudirman Kav. 31, Jakarta, 12920 Indonesia; 4 Associate Director, Water, Sanitation and Hygiene Section, UNICEF, 3 UN Plaza, New York, NY 10017 USA

**Keywords:** Sanitation, Health impact, Equity, SDG target 6, Wash, Indonesia

## Abstract

Recent evidence points to the possible underestimation of the health and nutrition impact of sanitation. Community sanitation coverage may first need to reach thresholds in the order of 60% or higher, to optimize health and nutrition gains. Increasing coverage of sanitation to levels below 60% of community coverage may not result in substantial gains. For example, moving Indonesia from 60% to 100% improved sanitation coverage could significantly reduce diarrhoea in children under 5 years old (by an estimated 24% reduction in odds ratio for child diarrhoea morbidity) with gains split equally by reaching underserved communities and the unserved within communities. We review the implications of these results across three levels of program implementation – from micro level approaches (that support communities to achieve open defecation-free status), to meso level (sub-national implementation) to macro level approaches for the national enabling environment and the global push to the Sustainable Development Goals. We found significant equity implications and recommend that future studies focus more extensively on community coverage levels and verified community open defecation free status rather than household access alone. Sanitation practitioners may consider developing phased approaches to improving water, sanitation and hygiene in communities while prioritizing the unserved or underserved.

## Background

Several recent studies [1–3] examine the impact of sanitation on health and nutrition and increasingly highlight the importance of achieving minimum levels of community improved sanitation coverage, i.e. critical thresholds, for achieving health and nutrition outcomes. A common finding is that the health and nutrition benefits of improved sanitation are only seen once a threshold of approximately 60% coverage or higher is achieved; exact threshold values may vary across studies. An impact on child stunting was found once a threshold of 75% community sanitation coverage was reached [4] while a similar value was suggested for health gains [5] while there are calls for further research into these threshold community sanitation coverage values [6].

This growing evidence is important for the design of sanitation programs. Sanitation practitioners remain puzzled by the previous underwhelming estimates of health impact from sanitation. These were based on cross-sectional regression analyses not considering the potential effects brought about by poor sanitation of a few households to the entire community, e.g. [7], or the small number of randomized control trials undertaken to date on this subject e.g. [8–10]. The new findings matter to those who deliver sanitation programs – from one side of the debate that questions whether communities might be better off focusing on interventions around water, handwashing, vaccination and clinical management of diarrhoea [11] to the other side flagging the inherent difficulties of measuring impact in water and sanitation interventions and the need to end open defecation in any case based on the best available evidence given that it is a basic human right [12]. The equity implications are clear, given the vast majority of the burden of poor water and sanitation still falls on the poorest [13]. These implications are further examined below at three levels (global, national and sub-national) and put into a sample country context with recommendations made for future work.

## Implications at the macro, meso and micro levels

Sanitation practitioners have tended to get on with the ‘how’ of accelerating sanitation rather than the ‘why’ but this debate on the health impact of sanitation matters on many levels and here we examine three such levels – from the macro level (global to national) to the micro level (at the community) and the critically important, but often overlooked, meso level in between – by meso we refer to the sub-national administrative units (Province or District level) where national policy is often expected to be implemented but without full understanding, capacity or resources being put into place to achieve this.

Multiple recent and complementary research findings resonate at the macro level where sanitation has often been a discounted component of the health sector; as stated in the past ‘the global health community is standing aside, absolving itself of responsibility, and firmly passing the buck to the water and sanitation sectors’ [14]. The new evidence suggesting the potential for serious underestimation of the health and nutrition impact of sanitation [e.g. 6] imply a strong need for renewed focus and funding for sanitation, particularly to reach the poorest. This is especially relevant for the Sustainable Development Goals (SDGs) where a considerable push is needed to ensure everyone has access to safely managed sanitation by 2030 [15] and substantial resources will be needed to achieve this [16]; sanitation is anyway a good investment purely in economic terms [17].

The debate, however, is equally important at the micro (community) level as the new evidence strongly reinforces the push to go beyond an incremental increase in toilet coverage but have people living in communities that are Open Defecation Free (ODF). This goal has gained global momentum through the Community-led Total Sanitation (CLTS) approach [18] or the Community Approaches to Total Sanitation (CATS) methodology [19] whereby communities support themselves to eliminate the practice of open defecation across the entire community through the creation of new social norms [20, 21]. Therefore, achieving ODF status would seem to complement well the emerging evidence that achieving minimum coverage thresholds are necessary to optimize health and nutrition impact.

To end open defecation by 2030, we need to accelerate progress and this would entail reducing the number of those practicing open defecation by 60 million per year, every year. The creation of social norms around latrine use at community level and achieving ODF is possible within a matter of months [18]. UNICEF have seen considerable and rapid progress in this respect in over 50 countries [19] across multiple regions and contexts. However, sustaining ODF status remains challenging and needs to be comprehensively addressed if countries are to maintain progress towards the SDGs [22, 23]. Supporting communities, especially the most disadvantaged and their most marginalized members, to sustain ODF beyond the initial celebration may need considerably longer timelines and will certainly need consistent monitoring. The process can also allow communities to broaden their focus beyond open defecation and expand into handwashing, safe storage of water, solid and liquid waste management etc. (for example this is the approach taken by Indonesia with the National Health and Hygiene program of the Ministry of Health, known as STBM) and even further into nutrition-related interventions and other behavioral change and developmental issues.

Using community approaches to achieve ODF communities can create new social norms and improve cooperation at community level [21, 24] but this may not be sustained without a longer and a broader perspective, e.g. [23, 25]. The new evidence reinforces that social norms are important not just for political will and community motivation but also to effectively impact on the negative health externalities of practicing open defecation. Indeed, herd protection accounts for a substantial portion of the total protection provided by sanitation interventions and many studies may be failing to account for these indirect effects and, thus, underestimating the impact of sanitation may be having [26].

The equity perspective of sanitation is clearly articulated in the SDGs given that poor sanitation hits the poor the hardest, especially the children living in the poorest households [27] where we see most reversion to open defecation. Therefore, we cannot advocate to reach 60% community sanitation coverage only as this would tend to favor the upper class and middle class segments of the community – the remaining 40% would tend to be clustered together in the poor areas bearing the brunt of the missing services [13] and contribute the most to the poor health and nutrition indicators [e.g. 12, 14, 15, 27].

## Example of these implications at a country level: Indonesia

**Fig. 1:**
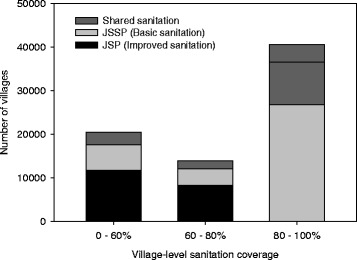
Village-level sanitation coverage (i.e. Improved sanitation (Jamban Sehat Permanen, JSP), basic sanitation (Jamban Sehat Semi Permanen, JSSP) and shared facilities combined) in Indonesia. Total number of villages = 75,017. Three types of color within each bar denote relative contribution of JSP, JSSP and shared sanitation facilities averaged in each village-level sanitation coverage category. Data source: STBM-SMS-based data, March, 2017

**Fig. 2:**
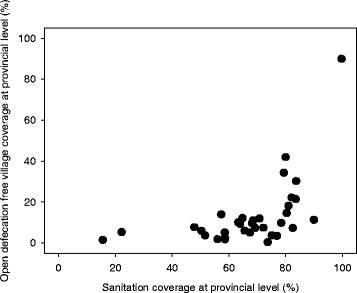
Sanitation coverage (i.e. Improved sanitation (Jamban Sehat Permanen, JSP), basic sanitation (Jamban Sehat Semi Permanen, JSSP) and shared facilities combined) vs ODF (verified and/or claimed) village coverage at provincial level. N Provinces = 34. Data source: STBM-SMS-based data, March, 2017

We take Indonesia as an example where these new findings may have significant implications. The country hosts one of the highest global populations practicing open defecation, over 30 million people [13]. At a national level it would appear that Indonesia has reached the threshold of over 85% total sanitation (improved and unimproved) coverage. However, the National Health Survey (2013) shows a total improved sanitation coverage of only 60% and no Province has over 80% coverage of improved sanitation; in terms of equity nearly all open defecation happens in the poorest 40% [28]. Though Indonesia has made remarkable progress with 54% of villages achieving higher than 80% sanitation coverage, it is also evident that over one quarter of villages are still at less than 60% coverage (Fig. 1) and thus not yet enjoying optimal health and nutrition impact. The provincial variations are even starker as only one Province has a substantial percentage of ODF villages even though some 24 Provinces have a sanitation coverage of over 60% (Fig. 2). It is clear from the new evidence that Indonesia has to move much more villages to the top right hand corner of Fig. 2 if the country is to lower its diarrhoea and stunting rates.

The prevalence of under-five diarrhoea morbidity in Indonesia was estimated as 14.3% in 2012, ranging from 10.4% in the richest to 16.9% in the poorest quintiles [29]. Applying the exposure-response curve of diarrhoeal morbidity and neighborhood fraction of improved sanitation, as modeled by Jung et al. [1] and using the village-level fraction of improved sanitation coverage and the proportion of villages in each 10% increment sanitation coverage category for odds ratio (OR) estimation, it is evident that achieving universal access may significantly reduce child diarrhoea morbidity in Indonesia. The analysis found, under a scenario of all Indonesian communities reaching ODF, then a total reduction of 23.7% in the odds ratio (i.e. from OR 0.67.7 to OR 0.44 with baseline reference of 0% coverage) of children under 5 diarrhoea morbidity can be achieved over the current situation. Given that a quarter of villages in Indonesia have less than 60% sanitation coverage, reaching these underserved communities is a priority to tackle the current unequal distribution of the diarrhoea burden; getting these communities to ODF status would account for half of the potential gains alone. The other half of the potential gains in childhood diarrhoea averted comes from the unserved within communities currently at over 60% sanitation coverage. Therefore, strategies for reaching both the unserved within communities and underserved communities are equally needed.

The Government of Indonesia is working hard on the stubbornly high levels of stunting which poor water and sanitation has been shown to clearly impact upon [30]. It would appear that accelerating STBM, the national sanitation and hygiene program, is key to addressing this and in doing so help reduce current inequities; for example stunting rates in the poorest 40% of Indonesia are 50% higher than in the richest 40% [28] though universal sanitation coverage would help all children in the community, especially in the poorest families.

## Nurturing the enabling environment

**Fig. 3:**
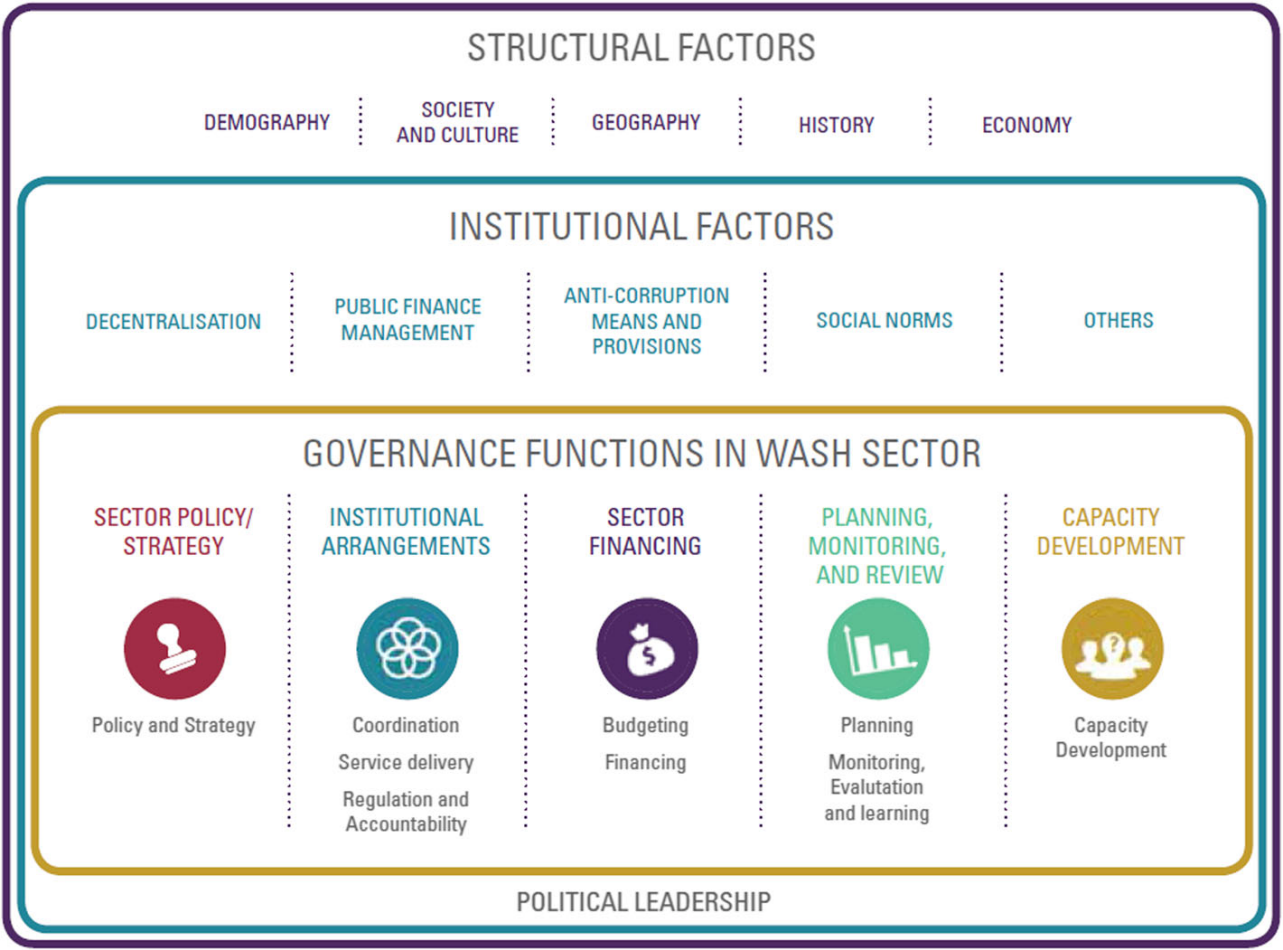
Contextual Factors Surrounding Enabling Environment Functions, from [22]

The creation of a strong Enabling Environment [31] is critical for achieving the required thresholds of community sanitation at scale. The Enabling Environment can be seen as an inter-connected set of functions and players that determine if a country, or a sub-national subset, can achieve quality, equitable and sustainable sanitation services at scale (Fig. 3). This is especially important at the meso level where national policies are passed down to a Province or District administration in the expectation of implementation, but often without a shared understanding of the policy, the required human capacity to roll it out, or guidance on how it needs to be resourced, implemented and monitored. A strong enabling environment and improved government efficacy can impact on other environmental health interventions also, for example water safety, fecal sludge management, solid waste and even issues beyond water and sanitation. Universal sanitation coverage has to be built upon a solid foundation involving the full range of elements of the Enabling Environment of Figure with a phased approach to get a country to universal sanitation and beyond, as per SDG Target 6.2.

## Looking forward

Given the clear and significant impact that sanitation has on health and nutrition outcomes, Ministries of Health around the world should be urgently discussing on how to raise community sanitation coverage to at least 60% and ideally to 100%, irrespective of where the responsibility for sanitation lies.

Stunting is central to this discussion; stunting impacts were reported in villages in Mali with higher sanitation coverage (reported latrine ownership by households was 64% with the other 36% sharing) though no impacts on diarrhoeal morbidity were described [10]. In that study it is interesting to note that although less than 10% of adults reported practicing open defecation, over 40% of children continued to do so.

When coupled with other additional adverse impacts (also probably underestimated) on school retention [32] and gender and dignity [33] it is clear that SDG Goal 6.2 is one that the world cannot afford to miss if other SDG goals on health, nutrition, education and gender are also to be achieved. A word of caution, from an equity perspective, for countries in this respect would be to keep a strong focus of this SDG tracking on the disaggregated progress around the elimination of open defecation in communities and not solely on increasing the safely-managed sanitation aspects. Countries should not increase progress towards the SDG 6.2 by solely enhancing access to safe faecal sludge management in already-served communities (or sections of them) but must prioritize increasing the coverage of basic sanitation where most needed; preferably to achieve completely ODF communities and so benefiting the underserved the most. The meso level has an important role to play here in terms of developing phased planning approaches to cover the duration of the SDGs, from now up to 2030.

There is also a need for greater emphasis to be placed by the meso level on monitoring of the achievement of ODF communities as opposed to solely tracking household sanitation coverage. This would also help get around the recurring problem of little or no toilet usage; less than half of household members reported using their latrines all the time in villages with 72% mean latrine coverage in Orissa, India [34]. The introduction of objective indicators such as presence of human excreta in the community or signs of latrine usage, as opposed to focus on counting toilets, may help in getting and sustaining sanitation coverage at the levels required for health impact [e.g. 23]. National Government could support this meso level push for ODF and encourage more effective mechanisms for the transfer of operational learning across administrative areas as opposed to just providing ‘prescriptions about specific approaches to be used throughout the country’ [35].

## Conclusions

The recent emergence of multiple evidence indicating the importance of achieving threshold values of community sanitation for health and nutrition outcomes are of immense significance for sanitation programming, from global to community level. Based on this, the following key implications are presented:

1. Previous studies may have underestimated the impact of sanitation interventions by focusing on household toilet coverage levels rather than overall community usage of sanitation. We recommend that future studies focus more on achievement of entire community coverage data and verified community open defecation free status.
2. The findings supports SDG 6.2 and justifies the push to eliminate open defecation using community approaches that aim to create strong social norms around creating and sustaining ODF communities. This can assist countries to steer clear of only looking to increase progress by first prioritizing the ‘safely managed’ aspects of existing sanitation systems. This is critical from an equity perspective as it means prioritizing the unserved or underserved over improving the lot of the already served – phased approaches at the meso level can help in this respect. Universal sanitation coverage benefits everyone, but with the poor benefitting more as they currently shoulder most of the health and nutrition burden of open defecation.
3. ODF communities are achievable, and in timelines of months, though we do recognize that as national sanitation programmes mature further and expand at subnational or national scale then additional complexities such as differences in the enabling environment, geophysical and social heterogeneity require more nuanced and well-resourced (financial and human capacity) interventions.
4. We believe that ODF achievement can steer communities and governments to take on, building on the strong social norms created at community level during the ODF process, other challenges such as water safety and solid waste management; phased approaches over a multi-year timeline can support this.

Finally, even if health and nutrition impact may be achieved at threshold values of around 60% community sanitation coverage, from equity, dignity, gender and sustainability perspectives we should still strive for not less than open defecation free communities. As countries prepare their SDG plans of action this salient point should be well reflected into national sanitation policies and planning.
